# Direct connections assist neurons to detect correlation in small amplitude noises

**DOI:** 10.3389/fncom.2013.00108

**Published:** 2013-08-14

**Authors:** E. Bolhasani, Y. Azizi, A. Valizadeh

**Affiliations:** Department of Physics, Institute for Advanced Studies in Basic SciencesZanjan, Iran

**Keywords:** correlation, correlation transfer, coupling, inhomogeneity, synchrony

## Abstract

We address a question on the effect of common stochastic inputs on the correlation of the spike trains of two neurons when they are coupled through direct connections. We show that the change in the correlation of small amplitude stochastic inputs can be better detected when the neurons are connected by direct excitatory couplings. Depending on whether intrinsic firing rate of the neurons is identical or slightly different, symmetric or asymmetric connections can increase the sensitivity of the system to the input correlation by changing the mean slope of the correlation transfer function over a given range of input correlation. In either case, there is also an optimum value for synaptic strength which maximizes the sensitivity of the system to the changes in input correlation.

## 1. Introduction

The recent advent of novel recording techniques has made it easier to simultaneously record from a large number of neurons and has provided new possibilities to relate population activity to coding and information processing in the brain (Greenberg et al., 2008; Cohen and Kohn, 2011). Many researchers suggest that studying the correlated activity of neurons in a population is essential for understanding how information is coded in the brain (Zohary et al., 1994; Abbott and Dayan, 1999; Nirenberg and Latham, 2003; Averbeck et al., 2006; Biederlack et al., 2006; Schneidman et al., 2006; Pillow et al., 2008). Correlated spiking of neurons contributes in several cognitive functions such as attention (Steinmetz et al., 2000), sensory coding (Christopher deCharms and Merzenich, 1996; Bair et al., 2001; Doiron et al., 2004; Galán et al., 2006; Schoppa, 2006) and discrimination (Stopfer et al., 1997; Kenyon et al., 2004), motor behavior (Maynard et al., 1999) and population coding (Sompolinsky et al., 2001; Averbeck et al., 2006; Josic et al., 2009). In addition to the functional effects of such correlations between populations of neurons on neural coding, understanding how different parameters such as biological, network or stimulus parameters tune them is eventually being revealed (Shadlen and Newsome, 1998; Binder and Powers, 2001; Moreno et al., 2002; Moreno-Bote and Parga, 2006; Tchumatchenko et al., 2010b; Rosenbaum and Josić, 2011b). Correlation between neuronal activities is measured frequently by pairwise correlation coefficients and spike count correlations, and the ability of a neuronal system to transfer correlation can be quantified by the correlation transfer function (CTF), which determines the relation between the output correlation of a system under stimulus and a specific input correlation (Doiron et al., 2006; Shea-Brown et al., 2008; Rosenbaum and Josić, 2011b).

A periodic common input on two (or more) uncoupled oscillators can cause coherent behavior when both oscillators lock to the external force (Pikovsky et al., 2003). A very common example is the control of circadian rhythms of humans/animals by the light-dark stimulation (Roberts, 2005). In case of noisy inputs the counterpart of the phenomena appears as stochastic synchronization (SS) which is a general topic that addresses the phenomenon of irregular phase locking between two noisy non-linear oscillators (Neiman et al., 1999). In nervous systems, cross-correlations can arise either from the presence of direct synaptic connections (Csicsvari et al., 1998; Barthó et al., 2004;) or from shared inputs from the surrounding network or sensory layers (Binder and Powers, 2001; Türker and Powers, 2001, 2004). Effect of direct synaptic connections and common inputs have been widely studied, but these two sources of correlation can be present concurrently in many physical and biological systems and their interplay can result in quite interesting phenomena. Couplings can regulate the activity of noisy oscillators and less variability in neuronal dynamics emerges through synchronization in networks of coupled noisy oscillators (Ly and Ermentrout, 2010; Tabareau et al., 2010; Zilli and Hasselmo, 2010). Studies on the correlation of spike trains have reported increase and decrease of correlation due to the presence of excitatory and inhibitory synapses, respectively (Rosenbaum and Josić, 2011a; Ly et al., 2012). When delay in communication and type of excitabilty of neurons are taken into account, the generality of these results can be debated since both excitatory and inhibitory synapses can be sources of synchrony and may increase correlation in different parameter ranges (Vreeswijk et al., 1994; Wang et al., 2012; Sadeghi and Valizadeh, 2013). Regarding the type of excitability and categorizing couplings as synchronizing and desynchronizing, it has been shown that shared inputs and direct couplings can show cooperative or disruptive effects on the correlation of noisy coupled oscillators (Ly and Ermentrout, 2009).

Possible differences between intrinsic parameters of neurons causes the message from the environment to the system to be decoded differently by the system components. Another aim of the current study is to investigate how the correlation is transferred by two neurons when the neurons are not identical. In such a heterogeneous system, the temporal symmetry of spike correlation is lost (Tchumatchenko et al., 2010b). We will show that with small amplitude stochastic inputs, even a slight inhomogeneity in the intrinsic parameters can lead to a large reduction of the pairwise correlation coefficient in the case of uncoupled neurons. As expected, the results depend on the time bins over which the correlation is calculated: spike count correlations over long time bins are less affected by the heterogeneity but synchrony—alignment of the action potential in small time bins—is tightly dependent on the homogeneity of the system.

We have shown that correlated inputs and direct connections can either show cooperative or disruptive effects in different ranges of parameters. For uncoupled neurons, correlation susceptibility increases by increasing the amplitude of noise for mildly correlated inputs (De La Rocha et al., 2007; Shea-Brown et al., 2008; Tchumatchenko et al., 2010b). We show that when direct connections are present between non-identical neurons, the mean susceptibility is not a monotonic function of the amplitude of the correlated noisy input anymore. Reminiscence of stochastic resonance phenomena, an intermediate noise amplitude in this case, leads to larger a sensitivity of the system to the changes in input correlation. We have also shown that with monosynaptic connections between two neurons, presence of inhomogeneity in the intrinsic firing rate of the neurons can enhance correlation of spike trains while for symmetric couplings, maximum correlation is seen for homogeneous system. Changing mismatch and synaptic strengths between two neurons, it is possible to change the functional form of the correlation transfer function to optimize the mean correlation susceptibility which is an indicator of the sensitivity of the system to the change of input correlation in different ranges. In this way, as the most important result of current study, we will show that with direct couplings it is possible to detect correlation in small amplitude noises by increasing the sensitivity of the system to the change of correlation in small amplitude noisy inputs.

## 2. Materials and methods

**Figure 1:**
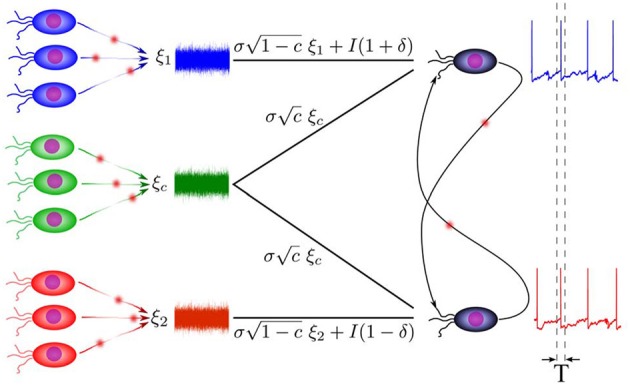
**Schematic representation of the model**. Two neurons stimulated by common and independent components, are possibly connected together by direct excitatory synaptic connections. Correlation of spike trains is then calculated over time bins much smaller than the mean inter-spike intervals.

The system under investigation consists of two coupled leaky integrate and fire (LIF) neurons (Knight, 1972), subjected to correlated stochastic inputs (see Figure 1). Subthreshold dynamics of the LIF neuron obeys the following first order equation:

(1) $$\tau_{m}\frac{dv_{i}}{dt}=V_{\text{rest}}-v_{i}+I_{i}+I_{ij},$$

in which *v*_*i*_ is a voltage-like variable for each neuron labeled by *i* = 1, 2 with τ_*m*_ = 20 ms and *V*_rest_ = −70 mV. A severe non-linearity is imposed on the model by considering a threshold value *v*_*th*_ = −54 mV. Whenever this value is reached, the neuron *spikes* and the voltage resets to *v*_reset_ = −60 mV. [Parameters taken from Troyer and Miller (1997)]. The spikes of the neurons are recorded as *x*_*i*_(*t*) = ∑_*m*_ δ(*t* − *t*^*m*^_*i*_) where *t*^*m*^_*i*_ is the time of *m* th spike of the neuron *i*, and δ(*x*) is the Dirac delta function.

Each model neuron receives a synaptic current through the direct connection from the other neuron *I*_*ij*_, and an external current *I*_*i*_ representing the sensory input or the effect of the surrounding networks. In the model equations, external current to the neuron *i* comprises a constant (dc) and a stochastic component with amplitude σ. The stochastic inputs are sum of a common component ξ_*c*_(*t*) and an individual component ξ_*i*_(*t*):

(2) $$I_{i}(t)=(1\pm \delta )I+\sigma \left[\sqrt{1-c}\xi_{i}(t)+\sqrt{c}\xi_{c}(t)\right],$$

where ξ_*c*_(*t*) and ξ_*i*_(*t*) are mutually independent Gaussian stochastic processes with zero mean and unit variance 〈ξ_*i*_(*t*)ξ_*j*_(*t*')〉 = δ_*ij*_δ(*t* − *t*'). The parameter *c* ∈ [0, 1] determines correlation of external currents which will be referred to as the input correlation. With the minimal model we used, inhomogeneity in the intrinsic activity rates is imposed by different constant currents which are chosen as *I*_1_ = (1 + δ)*I* and *I*_2_ = (1 − δ)*I*, where δ is referred to as the parameter of inhomogeneity. With non-zero δ the neurons 1 and 2 will be the high frequency (fast) and low frequency (slow) neurons, respectively. The currents are chosen suprathreshold (>14 mV) such that the neurons fire periodically at vanishing noise. Note that in this mean driven regime presence of small amplitude noise results in small jitters in firing times and a narrow distribution of interspike intervals.

Neurons are pulse coupled. The neuron *i* receives a pulse by the strength Δ_*ij*_ every time the neuron *j* fires, so the synaptic current in Equation 1 can be written as *I*_*ij*_ = Δ_*ij*_ *x*_*j*_(*t*) where the synaptic strength Δ_*ij*_ can be positive (excitatory) or negative (inhibitory). For convenience, we call the connections 21 and 12, the forward and backward connections, respectively. Although the external and synaptic inputs appear as currents, they are actually measured in units of the membrane potential (mV) since a factor of the membrane resistance has been absorbed into their definition.

Co-fluctuations in the activity of neurons are measured over a range of timescales (for a review see Cohen and Kohn, 2011). Spike count correlation is usually measured over the time scales from tens of milliseconds to seconds, while synchrony, that is almost precise alignment of the spikes, is measured over the time scale of the typical width of an action potential. It has been shown that spike count correlation over the small bins, bins of the order of one millisecond, can be largely determined by zero-lag conditional firing rate which quantifies exact synchrony (Tchumatchenko et al., 2010a). In this study we focus on synchrony, by describing spike counts and correlation coefficients in discrete bins of duration *T* = 0.5 ms. Correlation coefficient of spike counts *n*_*i*_(*t*) = ∫^*t* + *T*^_*t*_*x*_*i*_(*s*)*ds*, is defined as the zero lag cross-correlation between *n*_1_ and *n*_2_:

(3) $$\rho_{T}=\frac{\langle n_{1}(t)n_{2}(t)\rangle -\langle n_{1}(t)\rangle \langle n_{2}(t)\rangle }{\sqrt{\langle n_{1}{\left(t\right)}^{2}\rangle -{\langle n_{1}(t)\rangle }^{2}}\sqrt{\langle n_{2}{\left(t\right)}^{2}\rangle -{\langle n_{2}(t)\rangle }^{2}}}.$$

Dependence of the output correlation to the input correlation shows how correlation is transferred along neuronal layers in the nervous system (Rosenbaum and Josić, 2011a). With varying input correlation while other parameters are fixed, we compute ρ_*T*_(*c*), correlation of spike trains as a function of input correlation. To study sensitivity of correlation of output spike trains to the change of input correlation, we use *mean correlation susceptibility* (MCS), the mean slope of ρ_*T*_(*c*) in a given range of *c* ∈ [*c*_1_, *c*_2_]:

(4) $$S_{T}(c_{1},c_{2})=\frac{\Delta \rho_{T}}{\Delta c}.$$

which shows ratio of the change of correlation of spike trains Δ ρ_*T*_ = ρ_*T*_(*c*_2_) − ρ_*T*_(*c*_1_) to the change of input correlation Δ *c* = *c*_2_ − *c*_1_. For two identical neurons with no direct connection, this value is equal to one when it is evaluated over the full range of input correlation [0, 1].

## 3. Results

**Figure 2:**
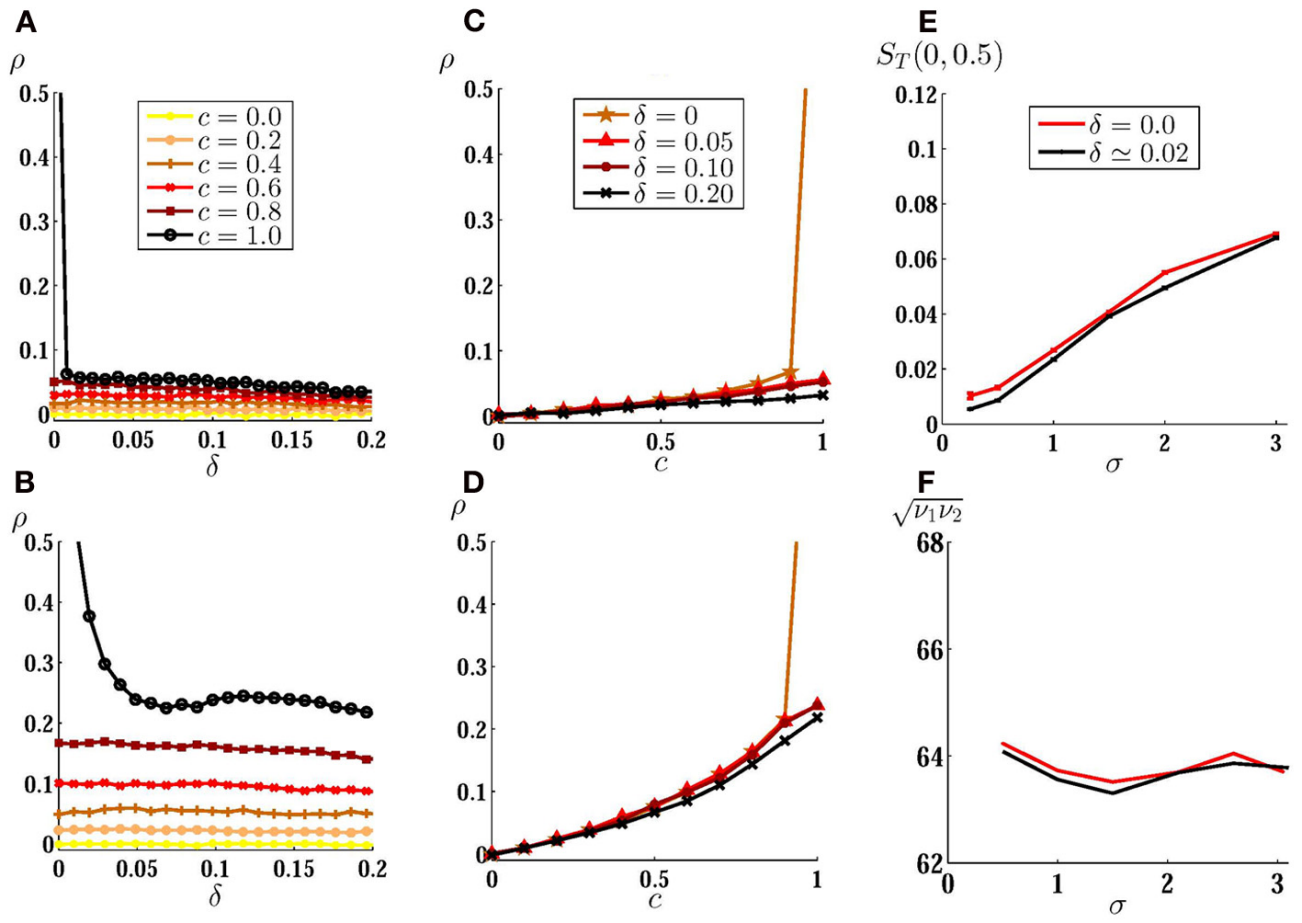
**Correlation of spike trains for two uncoupled neurons. (A)** Correlation coefficient is plotted against inhomogeneity, the mismatch between input current of neurons, for different values of input correlation and low noise amplitude σ = 1 mV. In **(B)** the same results are shown for larger value of noise amplitude σ = 5 mV with the same mean firing rate as **(A)** (see materials and methods). **(C,D)** Correlation transfer function, which shows the dependence of correlation of spike trains to the input correlation, is plotted for different values of inhomogeneity for the same noise amplitudes as **(A)** and **(B)**. **(E)** Mean correlation susceptibility (MCS) is plotted for homogeneous and slightly inhomogeneous systems, as a function of noise amplitude, which shows the mean sensitivity of the output correlation to the change of input correlation over the range [0, 0.5]. In **(F)** the geometric mean of the firing rate of the two neurons is shown when σ is varied.

We first present the results for two uncoupled neurons. In Figure 2A we have shown the cross-correlation coefficient as a function of the mismatch between intrinsic firing rates of neurons for low noise amplitude and different values of the input correlation. When there is no direct connection between the neurons, highly correlated inputs lead to a large output correlation in case of identical neurons. Even a small mismatch decreases the output correlation considerably if the noise is small amplitude. In this case, even common noises lead to a relatively low output correlation in the presence of a slight inhomogeneity (e.g., δ = 0.01 in Figure 2A). For larger noise amplitudes, the output correlation is less sensitive to inhomogeneity (Figure 2B). The system is also less sensitive to inhomogeneity when the inputs are weakly correlated where both homogeneous and inhomogeneous systems show a small output correlation. In Figures 2C,D we have shown the correlation transfer function. It can be seen that while the slope of the correlation transfer function decreases with mismatch for all the values of input correlation, this dependence is only noticeable when inputs are highly (completely) correlated. Increasing the noise amplitude (while decreasing the constant input to avoid a change in the mean firing rate as explained below) makes the output correlation less sensitive to inhomogeneity, yet the maximum sensitivity to mismatch is observed for highly correlated inputs (Figure 2D).

**Figure 3:**
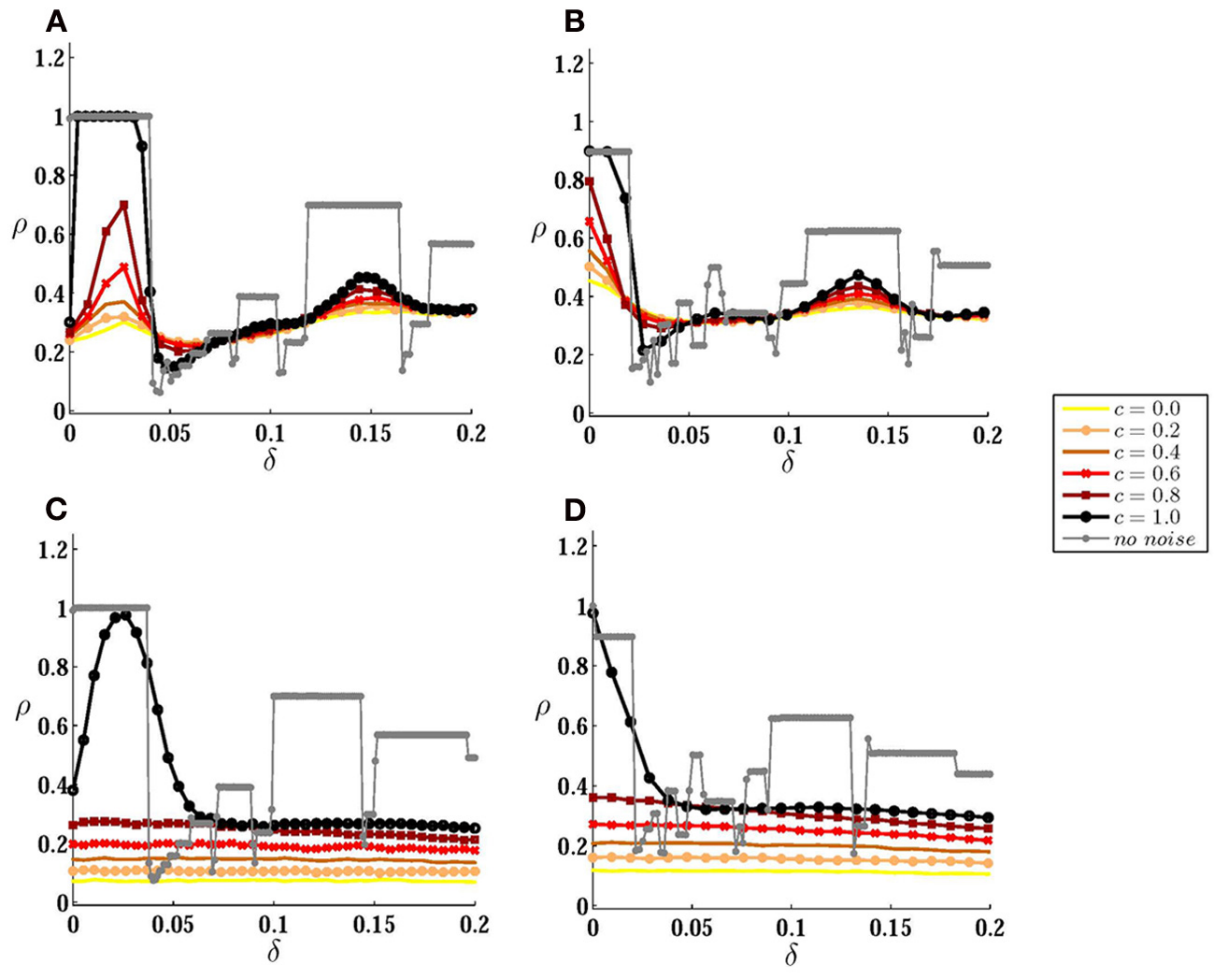
**Correlation of spike trains for coupled neurons. (A)** Correlation coefficient is plotted against inhomogeneity for different values of input correlation, when the neurons are connected by a forward excitatory connection (from the high-frequency to the low-frequency neuron) of the strength Δ_21_ = 1. **(B)** The same results are shown when the neurons are bidirectionally coupled by symmetric connections. In **(C)** and **(D)** the results are presented for larger noise amplitude σ = 5 mV. Noise amplitude in **(A)** and **(B)** is σ = 1 mV. The gray curves correspond to autonomous case when no stochastic input is present.

To show how sensitive are the correlation of spike trains to the input correlation, in Figure 2E we have plotted MCS (mean slope of ρ_*T*_(*c*) as described in materials and methods) as a function of the amplitude of the stochastic input for two uncoupled neurons over the range *c* ∈ [0 − 0.5] for homogeneous (δ = 0) and slightly inhomogeneous (δ = 0.02) systems. The system shows low sensitivity to the change in input correlation for small amplitude noises and the sensitivity smoothly increases with noise amplitude. Also, the presence of inhomogeneity has negligible effect on the mean correlation suceptibility: as noted above, for uncoupled neurons effect of inhomogeneity is only significant when inputs are highly correlated and while MCS is calculated over a range of weakly correlated inputs, it is almost insensitive to small inhomogeneity. While increasing the amplitude of the fluctuations, we have decreased mean value of the input currents to keep the mean firing rate almost constant (~64 Hz) as is shown in Figure 2F. In such a way the results observed in Figure 2E can not be attributed to the increase in firing rate which is known to increase the spike train correlation (De La Rocha et al., 2007; Shea-Brown et al., 2008). These results show that the correlation in small amplitude noises can not be suitably detected by a system of uncoupled neurons, whether the neurons have equal firing rates or their firing rates are different. To investigate the effect of direct couplings we have first considered a two neurons motif with just one unidirectional excitatory synapse. In many cases this configuration is favored when the synapses change through spike timing-dependent plasticity (Song et al., 2000). We considered an excitatory forward coupling from the high frequency neuron (as the presynaptic) to low frequency neuron (as the postsynaptic). In the absence of noise, any finite value of the forward coupling strength can lead to a zone of 1:1 synchrony, in which the dissimilar neurons fire in a causal master-slave fashion (Takahashi et al., 2009; Bayati and Valizadeh, 2012). In such causal limit the postsynaptic neuron fires immediately after receiving presynaptic stimulation (Woodman and Canavier, 2011; Wang et al., 2012). In our model delays in communication have been ignored, so in the causal 1:1 synchrony zones the postsynaptic neuron fires just one simulation time step after the firing of presynaptic neuron. Since the time bin on which the correlation is calculated contains several time steps (see materials and methods), such a causal master-slave firing leads to ρ = 1 (gray curves in Figure 3).

Stochastic inputs have non-trivial effects on the correlation of the spike trains of these two neurons. The output correlation is not a monotonically decreasing function of mismatch anymore, and in the presence of noise a small mismatch can increase the output correlation (Figure 3A). With zero mismatch, in the presence of one excitatory connection from neuron 1 to neuron 2 and in the absence of noise, the only stable state is the phase locked state in which neuron 2 fires one time step after neruon 1 (Bayati and Valizadeh, 2012). In the presence of noise this state looses stability as follows: because of the initial phase difference between the two neurons after master-slave firing (even though the phase difference is very small, just one time step), they respond slightly differently even to common noises. The different responses of the two neurons lead to a cumulative phase difference and if this phase difference results in the firing of neuron 2 before neuron 1 reaches threshold, the excitatory pulse from neuron 1 would be desynchronizing and makes the next firing of the two neurons further apart. The probability of the advancement of the phase of neuron 2 decreases in the presence of inhomogeneity (with *I*_1_ > *I*_2_), and with larger inhomogeneity it is less likely that the firing of neuron 2 (low frequency neuron) exceeds the firing of neuron 1 (high frequency neuron). When neuron 2 fires, before neuron 1 has reached the threshold, the excitatory pulse to the low frequency neuron will be synchronizing and if the voltage of neuron 2 is in the range [*v*_*th*_ − Δ_21_, *v*_*th*_] at the time of the firing of neuron 1, the neurons maintain causal master-slave firing. Further increasing the inhomogeneity lowers the probability of the voltage of the low frequency neuron reaching the range [*v*_*th*_ − Δ_21_, *v*_*th*_] at the time of the firing of the high frequency neuron, which results in the reduction of the spike trains correlation. A similar argument can explain the other notable rise and fall of the correlation which is seen in 1:2 locking zone of the noiseless system.

With symmetric bidirectional couplings, maximum correlation is obtained when the neurons are of the same firing rate (Figure 3B). When the neurons have equal firing rates (with *I*_1_ = *I*_2_) and in the absence of noise, each of the neurons can play the role of the master in a causal master-slave firing: in this case the connection from the master is synchronizing and the other connection has a desynchronizing effect (Bayati and Valizadeh, 2012). In the presence of small amplitude noise, the system can maintain causal locking by interchanging the role of two connections as synchronizing and desynchronizing. Suppose the firing of neuron 1 (master) is followed by the firing of neuron 2 (slave). Firing of neuron 2 exerts an excitatory pulse on neuron 1 but the phase advance of neuron 1 is relatively small because of the weak response of the LIF neuron at the beginning of its cycle (Mirollo and Strogatz, 1990). So it is probable that neuron 2 fires before neuron 1 reaches the threshold, then the excitatory pulse to neuron 1 would be synchronizing and neuron 1 fires immediately at the time it receives the pulse if its voltage is within the range [*v*_*th*_ − Δ_12_, *v*_*th*_] (note that the argument holds also in the presence of an absolute refractory period where the desynchonizing pulse from the slave neuron is ineffective). In the presence of inhomogeneity, it is the high frequency neuron that more probably plays the role of the master in a locked causal firing in the absence of the noise. In this case, in the presence of noise, inhomogeneity increases the probability that the voltage of low frequency neuron takes a value outside the range [*v*_*th*_ − Δ_21_, *v*_*th*_] at the time of the firing of the high frequency neuron, which reduces the correlation of spike trains as can be seen in Figure 3B for small values of inhomogeneity. For larger values of inhomogeneity, a bump can be seen again which belongs to the other main locking zone of the system in the absence of noise.

Intuitively, the relative amplitudes of noise and recurrent stimulations determine the behavior of the system and the most notable results can be expected when these two sources are of the same order, i.e., when neither the external noises nor recurrent stimulations are dominant. The results of Figures 3A,B are produced in this regime. For larger values of the noise amplitude, qualitative behavior of the system becomes more similar to the uncoupled system as shown in Figures 3C,D. For all partially correlated inputs, correlation of the spike trains is independent of the inhomogeneity and no signature of the locking zones is observed in the presence of large amplitude noises. It is only for common noise (γ = 1) that the effect of the unidirectional direct connection can be seen in the presence of strong noise in the region of the main locking zone.

**Figure 4:**
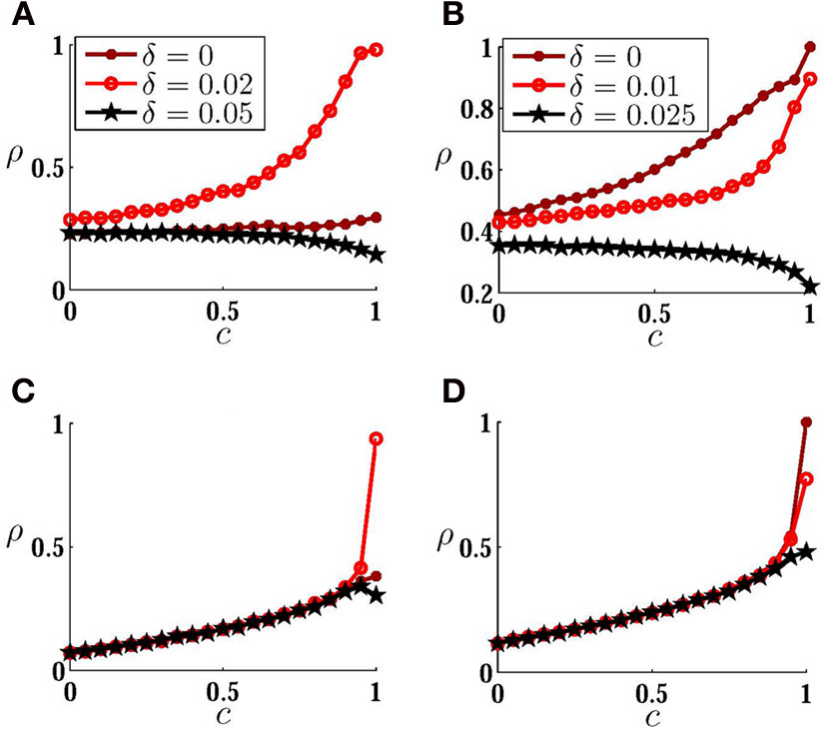
**Correlation transfer for coupled neurons. (A,B)** Correlation of spike trains ρ_*T*_ is plotted versus input correlation *c* for different values of inhomogeneity, when the neurons are connected by a forward excitatory connection **(A)** and by symmetric bidirectional couplings **(B)**. In **(C)** and **(D)** the results are presented for larger noise amplitudes. All the parameters are the same as those in Figure 3.

In Figure 4 we have plotted correlation of spike trains as a function of input correlation to inspect the effect of changing the correlation of the stochastic inputs on the correlation of the spike trains for a fixed value of the synaptic strength. When the noise amplitude is not large, depending on the mismatch, different dependencies of the output correlation to the input correlation can be observed (Figures 4A,B. Notably with changing mismatch it is possible to generate, for example, a system with higher sensitivity to the input correlation in different ranges of input correlation, or a negative slope ρ_*T*_(*c*). Comparing with the results of Figure 3 it can be deduced that high sensitivities on the input correlation is seen on the main locking zone (where the neurons are causally locked in 1:1 zone in the absence of the noise), and a negative slope is seen between two main locking zones. Again, as can be seen in Figures 4C,D, strong noises wash the signature of the direct couplings, and ρ_*T*_(*c*) for large amplitude noises is qualitatively similar to the uncoupled neurons.

**Figure 5:**
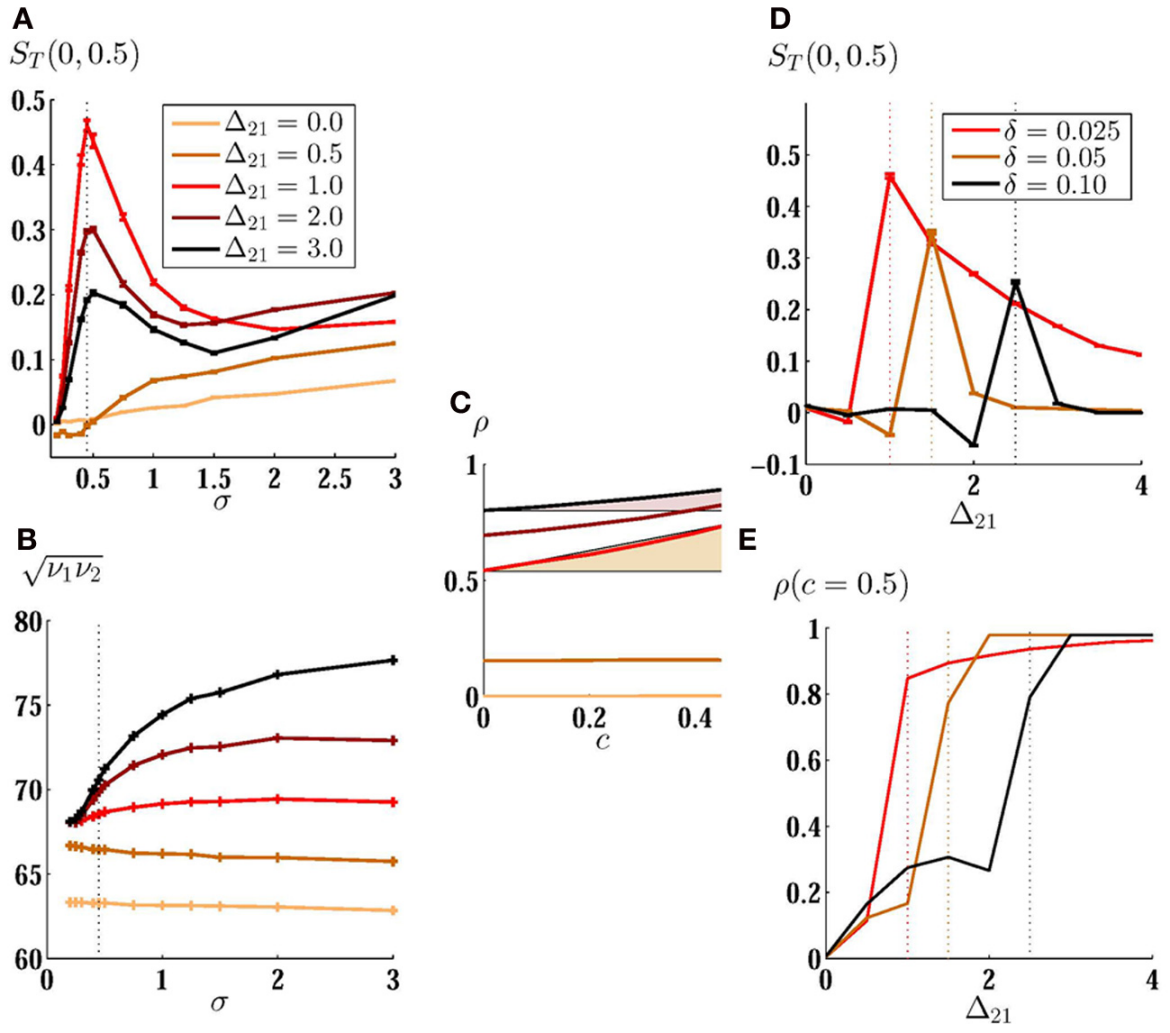
**Mean correlation susceptibility for coupled neurons. (A)** MCS is plotted versus noise amplitude for two unidirectionally coupled non-identical neurons (δ = 0.02). The results are shown for different values of synaptic strength. Maximum value of sensitivity to low amplitude noises can be obtained by Δ_21_ = 1. In **(B)** and **(C)** the firing rate of the neurons and ρ_*T*_(*c*) are shown for the corresponding curves in **(A)**, respectively. Shadings in **(C)** are guide to eye for a comparison of the mean slope of the ρ_*T*_(*c*) for two different values of synaptic strength. **(D)** MCS is shown as a function of synaptic strength for different value of mismatch. The optimum value for synaptic strength grows for larger mismatch. Correlation of the spike trains for *c* = 0.5 is shown in **(E)**. It can be seen that the correlation saturates when coupling constant is increased. Vertical dotted lines are plotted to show where the mean sensitivity is maximized.

Impact of direct connections on the detection of the input correlation of low amplitude noisy inputs is more apparent in a plot of MCS. In Figure 5A we have plotted *S*_*T*_(0, 0.5) as a function of noise amplitude for several values of synaptic strength, for unidirectionally coupled neurons and in the presence of a small mismatch in the intrinsic firing rates. As shown in Figure 5A, a forward monosynaptic connection (from high frequency to low frequency neuron) can considerably change the performance of the heterogeneous system in detecting variable input correlation. In an intermediate synaptic strength (Δ_21_ = 1) MCS shows a faster growth and a higher maximum in relatively small amplitude noise. Further increasing of the synaptic strength or the noise amplitude reduces the performance of the system in the detection of the input correlation. With very large noise amplitudes, the effect of the direct connections is washed out and all the curves, including that of the uncoupled neurons, merge together and the MCS smoothly increases with noise amplitude.

Overall increase of the correlation of the spike trains is an intuitive expectation when direct excitatory couplings are present in the systems (although this can be dependent on the type of excitability of the neurons). But how can direct connections increase the sensitivity to the changes in input correlation? In Figure 5B we have shown the geometric mean of the firing rate of the two neurons $\sqrt{\nu_{1}\nu_{2}}$ for the curves plotted in Figure 5A. Note that ν_2_ may be different from the intrinsic firing rate of neuron 2 because of the presence of an excitatory afferent synapse. The results show that the increase in the mean correlation susceptibility cannot be attributed to the increase of the mean firing rate of neurons, since then, larger coupling constants would lead to more sensitivity as they increase the mean firing rate of the system. A simple explanation can be found in Figure 5C: the degree of amplification of the output correlation depends on the input correlation. A suitable choice of the synaptic strength would result in more amplification for higher input correlations and would increase the slope of ρ_*T*_(*c*). Increasing the synaptic strength further, decreases the sensitivity due to the saturation of the correlation of the spike trains for the upper bound of the input correlation. In calculating MCS we have considered the range [0, 0.5] for the input correlation. Reducing the upper bound of this range increases the synaptic strength which saturates the correlation of the spike trains, so the synaptic strength which gives the maximum sensitivity increases with decreasing the range over which the mean sensitivity is calculated.

The *best* synaptic strength, which maximizes sensitivity, depends also on the mismatch between the intrinsic firing rate of the neurons as can be implicitly deduced from the results shown in Figures 3A,B. In Figure 5D we have shown MCS as a function of the strength of the forward unidirectional coupling for three values of mismatch. Optimum value of synaptic strength is larger when the intrinsic firing rate of the neurons are more different. Plots of the spike train correlation ρ for upper limiting value of the input correlation *c* = 0.5 again shows that the maximum mean sensitivity in this range is obtained when the spike train correlation is not saturated for the upper bound of the range of *c* (Figure 5E).

**Figure 6:**
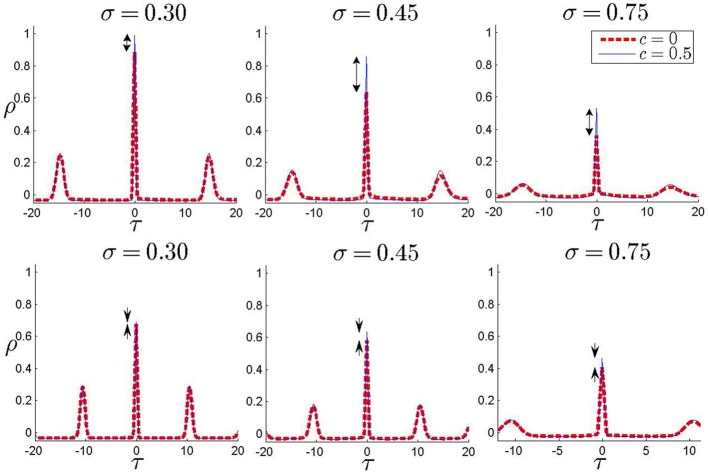
**Correlation coefficient for non-zero time lags**. In each panel, correlation coefficient is plotted against time lag for two values of input correlation *c* = 0 and *c* = 0.5. The results are shown for three different noise amplitudes (shown above each panel) and two different values of inhomogeneity parameter, δ = 0.02 in upper panels and δ = 0.15 in lower panels. One unidirectional connection of strength Δ_21_ = 1 is present from the high-frequency to the low-frequency neuron. The difference between two curves at zero lag gives MCS which has been shown in the plots by arrows.

All the results presented in this study have focused on the degree of zero-lag synchrony which is measured by the zero-lag cross correlation of the binned spike trains with small bin size. In the presence of an inhomogeneity and with asymmetric direct connections, it is possible that the maximum correlation of the spike trains appears in non-zero lag. In Figure 6 we have shown the cross-correlation coefficient of spike trains as a function of the time lag for three values of noise strength and two values of the input correlation (*c* = 0 and *c* = 0.5). It can be seen that the maximum cross correlation for all the values appears in zero time lag (more precisely at a time lag equal to one simulation time step). Presence of other maximums is an indicator of almost periodic firing of the neurons which arises from the suprathreshold mean and the small amplitude stochastic fluctuations of the input current. Results in Figure 6 are presented for one forward unidirectional coupling and sample values of inhomogeneity and synaptic strength. The results for other parameters are similar while the system is in the main locking zone in the absence of noise. This result shows a drawback of the simplified models we have used: LIF neurons with pulsatile instantaneous couplings can be synchronized with zero phase lag even in the presence of frequency mismatch, which is revealed as a maximum in correlation at zero lag (one simulation time step) when a small amplitude noise is added. Both mismatch and delay (synaptic and axonal) can be source of phase lag, when the neurons are modeled by limit cycle oscillators and more realistic models are used for synaptic currents. Our results are still valid when such phase lags are small, of the order of the time bins in the calculation of the correlation.

**Figure 7:**
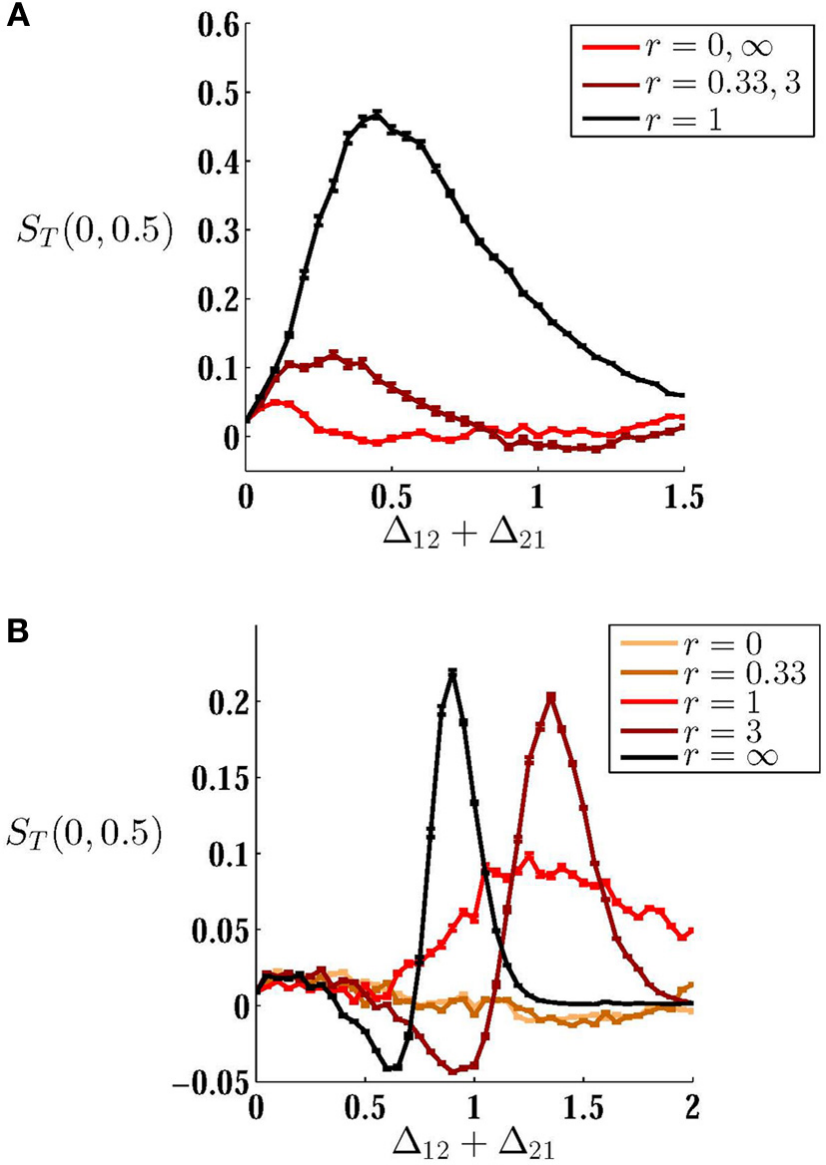
**How the configuration of connections affects sensitivity. (A,B)** MCS is plotted against sum of synaptic weights for **(A)** homogeneous system δ = 0, and **(B)** inhomogenous system δ = 0.1. Different curves are plotted for different ratios *r* of forward and backward couplings indicated in the legends. When the neurons have equal intrinsic firing rates, symmetric configuration *r* = 1 shows the best performance with a suitable choice of synaptic strengths. For inhomogeneous case when the imbalance of couplings is in favor of forward coupling (from the high frequency to low frequency neuron) the sensitivity is considerably improved. When the backward coupling is larger *r* < 1, the system performance is quite poor. As is shown in axes labels, MCS is calculated over the range [0, 0.5] of input correlation.

Above results were obtained for bidirectional symmetric couplings or for one unidirectional coupling. To find the *best* configuration through which direct couplings can improve the performance of the system in the detection of a variable input correlation, we have tested mutual couplings with different ratios of forward Δ_21_ and backward Δ_12_ connections. While the synaptic cost (sum of two synaptic strengths) is kept constant, different configurations can be designed by changing the ratio of the coupling constants *r* = Δ_21_/Δ_12_ (Figures 7A,B). In the absence of mismatch, the best configuration is that which preserves symmetry, i.e., the best performance results with equal forward and backward couplings. On the other hand, in the presence of mismatch, an asymmetric arrangement of couplings in which the forward coupling (from the high frequency neuron) is larger, improves the performance of the system. Interestingly, asymmetric excitatory couplings in favor of backward coupling (from the low frequency neuron), significantly decreases the sensitivity of the system since it plays the role of desynchronizing coupling as discussed above.

## 4. Discussion

Both direct connections and common inputs can be sources of the correlated activity of neurons in the nervous system. Effect of direct connections is widely studied as a general problem in dynamical systems and in particalur in nervous systems (Kuramoto, 1991; Strogatz and Mirollo, 1991; Abbott and van Vreeswijk, 1993). Stochastic inputs are usually a source of temporal disorder but spatial order can be induced in a neuronal pool when the neurons share stochastic inputs from common sources (Binder and Powers, 2001; Türker and Powers, 2001, 2004). Because of the possible cooperative/competitive effects of common inputs and direct connections, interesting results can be expected when they are concurrently present in a system (Ostojic et al., 2009; Ly and Ermentrout, 2010; Tabareau et al., 2010; Zilli and Hasselmo, 2010; Rosenbaum and Josić, 2011a; Ly et al., 2012). In this study we have numerically inspected the effect of correlated stochastic inputs on the correlation of spike trains of two coupled LIF neurons. We have mainly focused on the correlation of spike trains when correlated small amplitude noises were imposed on a system of two coupled neurons, and the neurons were regularly and synchronously firing in the absence of noise. We have shown that such a system shows high sensitivity to the changes of input correlation, and therefore can be a suitable detector of the correlation in small amplitude noises. To study the system in a more general framework, we have considered neurons with different intrinsic firing rates. We have assumed neurons have equal membrane time constants, and inhomogeneity is imposed on the system by feeding the neurons with unequal suprathreshold constant currents. The inhomogeneity, determined by the difference in the mean input currents, along with synaptic strengths are the key-parameters that specify the response of the system to stochastic inputs.

While for uncoupled neurons the output correlation is a monotonically decreasing function of inhomogeneity, for coupled neurons with low noise amplitudes, spike trains correlation can be increased by increasing inhomogeneity in some ranges. This result holds for sufficiently small noise amplitudes and the system inherits this property from *n*:*m* locking zones for the autonomous system when there is no stochastic input present. This introduces inhomogeneity as an important parameter with non-trivial impact on the correlation of spike trains in coupled systems.

Another feature of the system is that the two sources of correlation, correlated inputs and direct excitatory connections, do not necessarily cooperate in the formation of correlated spike trains. For uncoupled neurons output correlation is a monotonically increasing function of input correlation and for weakly correlated inputs, the slope decreases with lowering noise amplitude (De La Rocha et al., 2007; Shea-Brown et al., 2008) and with increasing mismatch. With different choices of the synaptic strengths and the inhomogeneity, it is possible to change functional form of correlation transfer (dependence of output correlation to the input correlation) and design a system with different sensitivity to the input correlation. In particular, it is possible to design a system with negative mean slope of correlation transfer, showing a case with destructive effect of common noises on the correlation of spike trains, or a system with maximum sensitivity to the changes in input correlation in a given range by maximizing the slope of correlation transfer. The latter proposes that direct connections can increase the sensitivity of the system to the correlation of the neuron's stochastic inputs, especially when the noises are small amplitude. We have further shown that for a homogeneous system (where the neurons have equal intrinsic firing rates), the best configuration of the couplings which maximizes the mean sensitivity of the system in a given range, is a symmetric configuration with equal coupling constants. On the other hand, in the presence of inhomogeneity, an asymmetric configuration in which the synaptic constant from the high frequency neuron to the low frequency neuron is larger, improves the sensitivity. In either case, there is an optimum value of the synaptic constant which maximizes the sensitivity.

Competitive learning through conventional spike timing-dependent plasticity (STDP) in feed-forward networks leads to the potentiation of the synapses which convey correlated data and depression of those with uncorrelated activity (Babadi and Abbott, 2010). How does STDP change the lateral connections transverse to the path of data flow? It has been shown that in the recurrent networks, asymmetric connections arise through STDP and in the presence of inhomogeneity, such an asymmetric change is in favor of the connection from the high frequency to the low frequency neuron (Takahashi et al., 2009; Bayati and Valizadeh, 2012). Our results show that asymmetric connections can enhance the performance of inhomogeneous systems in the detection of input correlation, and interestingly such an optimum configuration of connections emerges through STDP (with asymmetric profile) in inhomogeneous neuronal pools (Bayati and Valizadeh, 2012).

Type of neuronal excitability can also affect the correlation transfer in neuronal pools (Galán et al., 2008; Abouzeid and Ermentrout, 2009; Barreiro et al., 2010). Phase resetting curve characterizes how small perturbations influence the oscillator's subsequent timing or phase. It has been recently shown that uncoupled type-II neurons with both negative and positive regions in their PRC transfer correlations more faithfully when the correlation is calculated over short time bins (Abouzeid and Ermentrout, 2011). Since the phase of a LIF neuron always advances in response to the external pulses, the results for LIF neurons are likely to apply for type-I neurons.

Correlation of spike trains over such small time bins that we have used *T* = 0.5 ms, is a measure of (almost) precise alignment of the action potentials. Similar results were obtained when we repeated the experiments with *T* = 1 ms but we expect qualitatively different results when the correlation of the spike counts is measured over the time scales comparable, or larger than the mean inter-spike interval. Less sensitivity to the inhomogeneity is expected when the correlation is evaluated over large time bins, but the effect of direct couplings warrants further studies to find out if correlation in small amplitude stochastic inputs can be revealed in co-variation of spike trains of coupled neurons over large time scales.

### Conflict of interest statement

The authors declare that the research was conducted in the absence of any commercial or financial relationships that could be construed as a potential conflict of interest.
